# Supplementary figures and images for: Chromosomal Instability Causes Sensitivity to Polyamines and One-Carbon Metabolism
Source: Metabolites. 2023 May 9;13(5):642. doi: 10.3390/metabo13050642 (PMC10221085; doi:10.3390/metabo13050642)

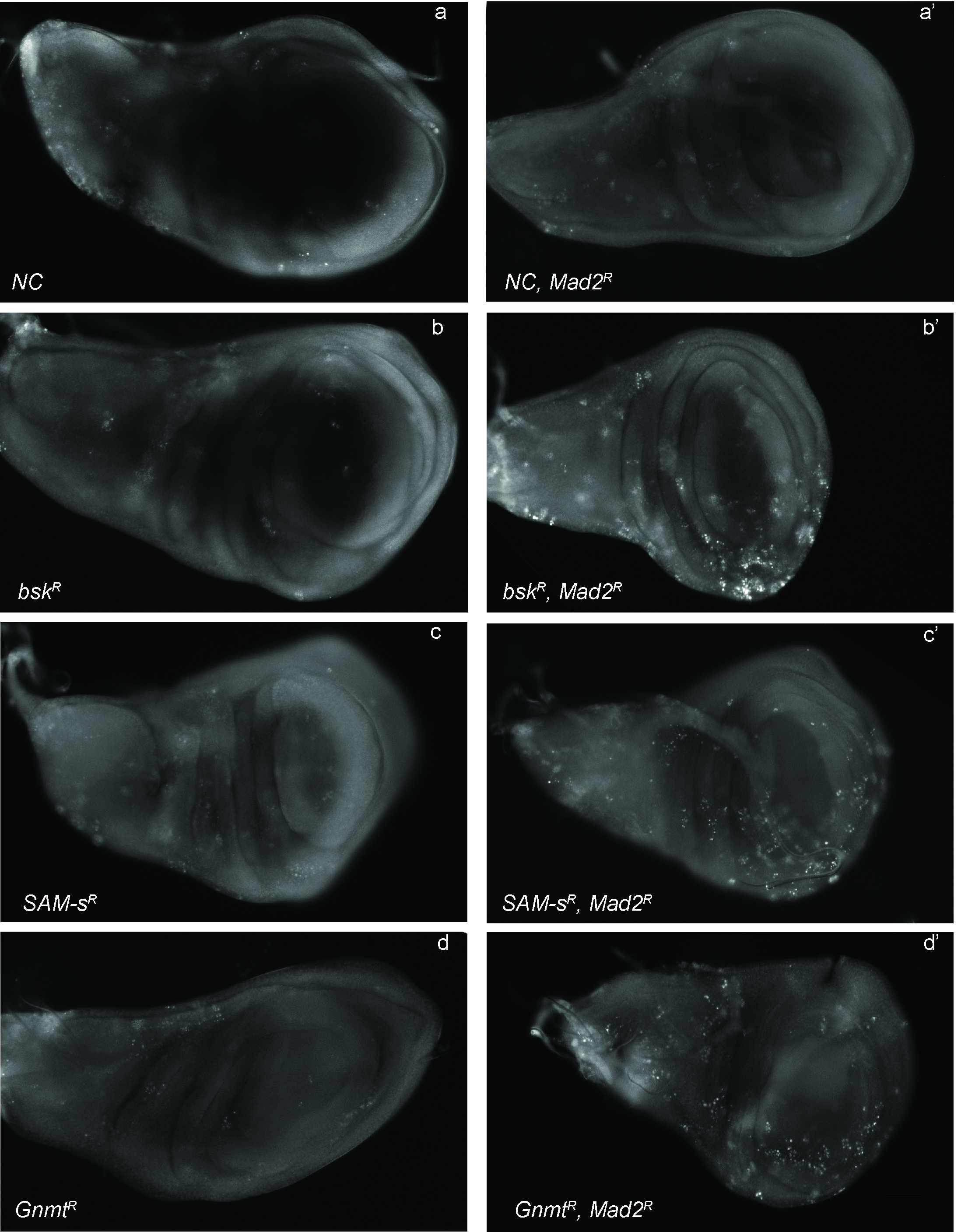

Supplement: Supplementary file 1 [file metabolites-13-00642-s001.zip › Supplementary Figure S1.jpg]

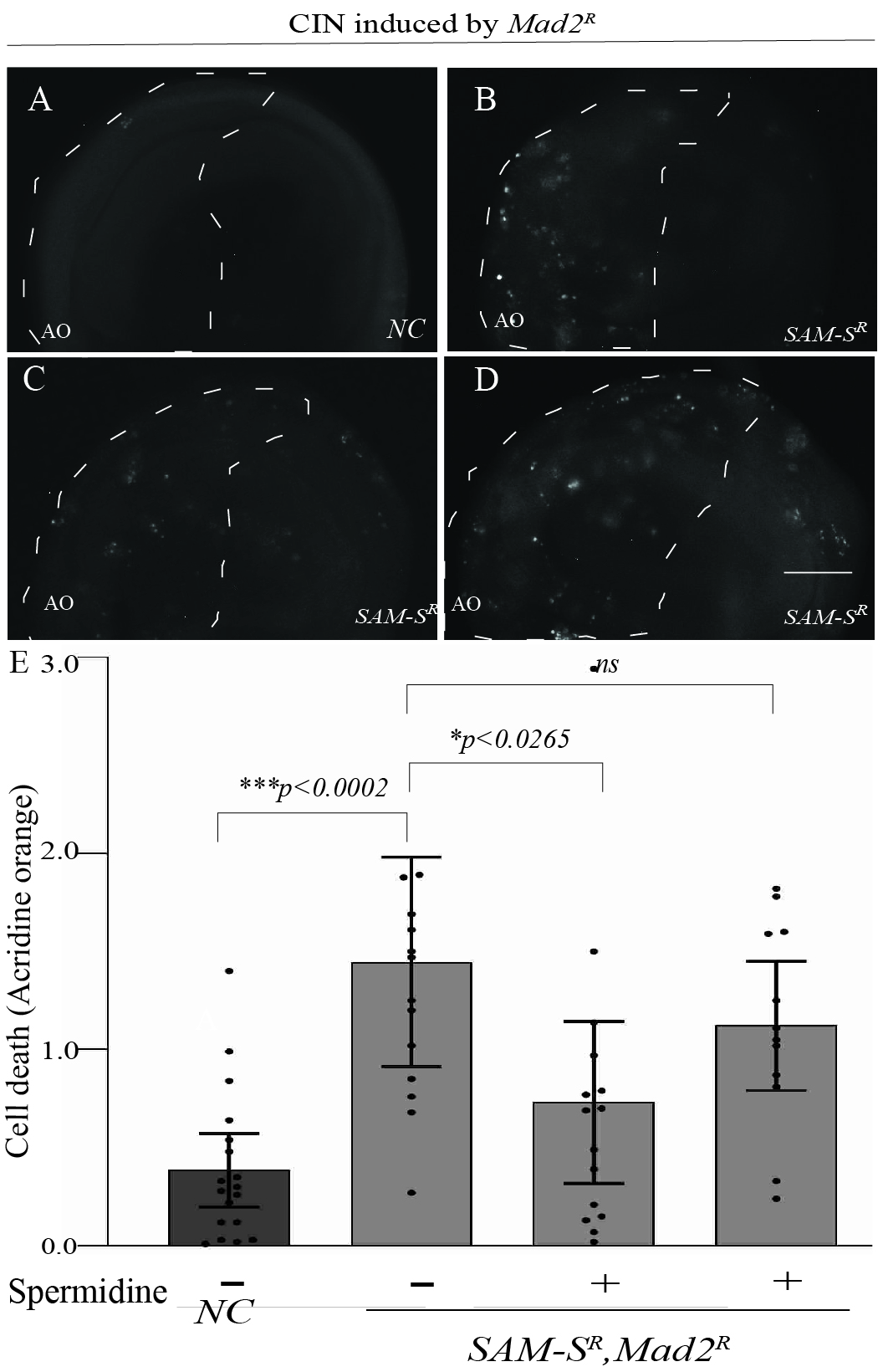

Supplement: Supplementary file 1 [file metabolites-13-00642-s001.zip › Supplementary Figure S2.jpg]
